# Dynamics of Metaverse and Medicine: A Review Article

**DOI:** 10.7759/cureus.31232

**Published:** 2022-11-08

**Authors:** Mrudul A Kawarase, Ashish Anjankar

**Affiliations:** 1 Medicine and Surgery, Jawaharlal Nehru Medical College, Datta Meghe Institute of Medical Sciences, Wardha, IND; 2 Biochemistry, Jawaharlal Nehru Medical College, Datta Meghe Institute of Medical Sciences, Wardha, IND

**Keywords:** augmented reality, virtual reality, research, medicine, simulations, metaverse

## Abstract

Metaverse is a relatively new concept of technological advancement for the world. Various sectors such as finance, entertainment, and communication are the forefront admirers of these innovations. Alongside these, the field of medicine has recently been on the list of metaverse-benefiting domains. Various aspects of medicine, such as educational and teaching purposes, surgical simulations, conferences and meetings, awareness programmes, research programmes, and many more, are under research. Depending on the requirement, the metaverse is a versatile platform which can be modulated accordingly, thus providing a flexible tool for medical development. In this review article, these domains are discussed in depth along with the pros and cons of the same, which positively affect the productivity of the field of medicine. All these headings have been through minimal study and experimentation, and the results obtained from them are satisfactory in terms of study. The primary purpose of the review article is to provide a suggestive statement regarding domains of the metaverse and their usage as a vital tool of operation in the future of medicine.

## Introduction and background

Metaverse, defined by Lee [1], is when the natural and virtual worlds form a coalition and carry out social, economic and cultural activities for value creation. Another definition is that it is a three-dimensional virtual reality in which routine and economic activities are conducted via virtual characters or avatars representing reality [2].

Metaverse is a concept which came to light in the late 1990s. Neal Stephenson was the first to use the word "metaverse" in his novel Snow Crash in the year 1992. In compilation, it refers to the reality beyond reality. It is a combination of the words “meta” and “universe,” which signify transcendence and virtuality, respectively. The digitized earth is a new world expressed through digital media such as cell phones and the internet, as defined by this concept [3]. After the notion of the metaverse became famous, much work and study made it a reality. In various development fields, the metaverse concept was introduced and is being extensively researched to date. Facebook, Amazon, Microsoft and many tech giants are pioneers in the significant developments in the metaverse usage and application field. Mainly the concepts of virtual reality and real-time connectivity were the core of concern.

Social media has considerable input in the metaverse building. Facebook has changed its name to Meta, showing its objective to transform the communication and information world into a metaverse [4]. The basis of the metaverse is blockchain which highlights the non-centralized nature of its existence without a third-party provider [5]. Thus with the emergence of cryptocurrency, the implementation of blockchain in the healthcare system has emerged as a promising feature [6]. Non-fungible tokens or NFTs are other modalities associated with the metaverse, which could be revolutionary. NFT is a one-of-a-kind, non-interchangeable packet of data registered in the blockchain and used as a token to assert ownership of a digital asset [7]. In medical emergencies, a person can save his/her personal information and medical records on an NFT. Instead of physically doing tests every time and waiting for external documents, this NFT can be shared with physicians whenever needed [8]. This is a fairly promising example to show that the usage of modalities provided by the metaverse has potential in the future development of modern medicine.

## Review

Educational improvement

Medical education is one of the many phenomena which are under extensive research and development regarding the improvement of the medical field from the very basic stages. The better the quality and delivery of education, the better the future of research and development of medicine. Conventional education has been in circulation for a long time, and productivity has slightly deteriorated because of the incompetence of the same in providing the quality and the magnitude of knowledge assessment and implementation. In examination conduction, applying a computer base is also a reforming concept. On January 6-7, 2022, the Korean Medical Licensing Examination (KMLE) was conducted via Computer Based Testing (CBT) [9]. The results obtained were better than the conventional methods.

 The current requirements are more towards the applied subjects and methodologies. Nevertheless, the research process and implementation are hampered due to the scarcity of resources in many places. The services provided by the metaverse include all the platforms of virtual connectivity and augmented reality tools for medical education. Medical education varies in place and time, which makes it different in different parts of the world. Metaverse would be able to provide a similar quality and standardized education regardless of the time and place [7]. Following are some of the domains of the metaverse which can be helpful in medical education. These domains could be viable solutions to the problems mentioned previously.

Augmented reality

Augmented reality can be considered an advanced and versatile version of the external world. The technology uses a location-aware system and interfaces as a source to expand the universe virtually and use it in applied fields of development [10].

In the case of medicine, augmented reality can be used as a tool for education and training as it provides a vast magnitude of knowledge, both textual and audiovisual. Simulation-based learning is the key feature of this provision. In this way, various case scenarios and conditions can be put forth to the learner so that the learner does not have to wait for that particular case to come across in the real world to study it [11]. A promising example to support it is Curiscope's Virtuali-Tee (Curiscope, Brighton, East Sussex, United Kingdom), which is an augmented reality t-shirt that gives the user the ability to see and examine the insides of a human body similar to that of an anatomy dissection laboratory [12].

Lifelogging 

Maintaining the records of research and even general medical practice is a major part of the process. These records include research findings, legal and fiscal records, patient information, etc. Conventionally, it is done via medical journals, physical papers, data servers, video/audio logs, etc. Even though these methods have proven efficient in these years, there is always a scope for innovation. Integration of different parts of record maintenance, thus increasing its efficiency, is the goal of lifelogging. Lifelogging is more likely a record-keeping tool in which medical professionals can record their work, such as case scenarios, surgeries, treatment regimen, and research projects, in a virtual format and even share it with others. An example is Classting Artificial Intelligence (AI) in Korea, an online connected learning community currently using lifelogging as a learning and teaching platform [13]. In this example, the learning community has combined record maintenance, sharing of data, and analysis, all in one convenient platform. 

Mirror world

In this type of augmented reality, real-life situations are simulated in the virtual world to create an experience-based learning environment. Digital laboratories and virtual educational centres are a few examples of the same, which help in creating a reflection of the natural world [10]. In medicine for example, in case of emergency situations like diabetic ketoacidosis where the window of learning is very narrow and the decisions are to be made fast as well as efficiently, which sometimes causes mistakes to occur, a relatively safer way of training medical professionals can be provided by the mirror world. These mirror worlds would provide simulations of emergencies to minimize the risk and maximize the learnings.

Almost all the provisions of the metaverse are based on the non-face-to-face (intact) principle, which can be accessed regardless of distance and space. This, in particular, has recently come into the limelight due to the COVID-19 pandemic. Significant sums of money are being invested by major technological corporations towards the development of the metaverse, whose key component will be the merger of the physical and virtual worlds [14]. The usage of virtual platforms such as digital labs has been a pioneer. At the University of Washington, David Baker and his team who studied the structure of proteins use the digital lab to find a structure of protein for an AIDS treatment. They devised a game in which various people would fold protein amino acid chains and the structure which matched to a close proximity would get points [15].

Virtual reality

This is the most popular type of multiversal modality as it comprises many interactive tools which can help in enhancing medical education. Three-dimensional (3D) graphics, avatars, and rapid communication tools are some of the most used tools in recent times. In this type of reality, the environment is built entirely from the real world, and thus the limitation of virtual reality is that person’s imagination [11]. Virtual reality metaverses such as Roblox (Roblox Corporation, San Mateo, California, United States) and Zepeto (Naver Corporation, Seongnam, South Korea) have been at the top of the list for providing a space for people who cannot get out of their homes due to the pandemic [16,17]. Meetings, VR sessions, simulations, tests and more are the various methodologies implemented to achieve the required goal.

**Figure 1:**
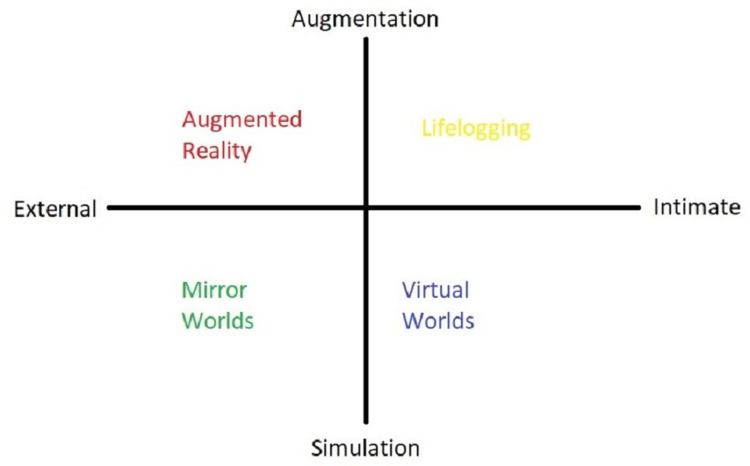
Components of the metaverse

Virtual reality can be used in simulation building, interactive learning, case scenario confrontations and many more such domains if the field of medical education is considered. Geographical and language barriers are the two main factors responsible for hampering sharing of knowledge worldwide, and with the help of technology like virtual reality, these factors can be omitted entirely from the equation of medicine. A pictorial representation of the components of the metaverse is given in brief in Figure 1.

Limitations 

Even though the metaverse has provided a large domain for the medical field to grow, the results obtained are still not as good as the real-world scenarios. Mainly three types of error are there due to which the quality of metaverse connectivity is affected. These are machine-generated errors, software errors, and human errors. Each one of these has a different impact on the process and results of virtual reality. For an extended period, among the domains of development, healthcare and life sciences have been one of the slowest in accepting technological advancements [18,19].

For example, suppose a population-based study is being conducted in a population in which AIDS is prominent. In that case, the most common errors that can occur if the virtual platform is used are incorrect data, insufficient data, calculation errors, and misinterpretation. Many factors can be responsible for such errors to occur such as human errors, equipment errors, security breaches, the occurrence of exceptions, etc. These kinds of errors occur in physical modes too, but the occurrence rate is shallow compared to the virtual one. As technology advances, both the required software and hardware will be available to shorten the magnitude of these errors. Another major limitation is the cost of the infrastructure building and training of the necessary staff, both monetary and energy-related, as a large amount of energy consumption is anticipated to sustain a viable metaverse [20]. Data breaches and security and privacy concerns due to a massive amount of personal data being shared are maximum [21].

Mental health

Mental health has been an essential aspect of human life, and the effects on mental health and the results of those effects on the same in total human productivity have been a topic of study in medicine for a long time. Since the metaverse is a relatively newer concept and its popularity has just begun to take place, it will pose its pros and cons on the users’ mental health. Even with all the pros and cons, it can b considered a promising tool for treating patients dealing with mental health issues. An excellent example of this is that in some studies, it has been seen that the mental health of healthcare professionals has been improved due to the usage of virtual reality interventions since they pose almost negligible occupational hazards [22]. Recently more advances are being made in the sector of mental counselling. Online counselling or tele counselling has recently surfaced in professional mental care [23,24].

Virtual reality therapy or VR therapy and other virtual counselling methodologies are in development to make the therapy session experience resemble reality more. In previous studies, it has been shown that these VR sessions have been helping workers in Japan cope with various mental traumas and psychological illnesses, which are based on Scholastic Assessment Test (SAT) counselling [13]. A suitable metaverse that can provide two-way communications and VR sessions closest to real-life experience will be able to deliver counselling and therapeutic services with more ease and of better quality. This can be considered a suitable methodology in some cases, as a virtual neuropsychological evaluation was conducted to confirm the same based on prior studies [25].

Audiovisual therapy

Audiovisual methods as a treatment alternative have been extensively used in recent years in managing mental illnesses such as anxiety and depression. This alternative method has surfaced due to many factors posed by conventional drug treatment and counselling. Incompetence, side effects, recurrence, and non-pocket friendly, are some of the factors responsible. The induction of audiovisual therapy can be done more easily with the help of metaversal provisions.

In some prior studies, it has been seen that music therapy, when added to the treatment plan along with drugs and counselling, can help patients affected by mental illnesses such as major depression. The comorbidities such as anxiety have also shown signs of improvement as these conditions are closely related. The most essential and promising factor of audiovisual therapy is that it poses minimal to negligible side effects compared to conventional treatment modalities. This therapy’s primary mechanism of action is that it reduces the severity of the actual condition’s signs and symptoms, which act complementary to medication, resulting in faster recovery and better quality of post-recovery period [26].

The metatarsal outlook of this concept is that with the help of augmented reality and VR, it will be easier to induct such kind of treatment as the base of virtual reality is majorly audio and video projecting sources. Experimental therapies can be efficiently conducted with the metaverse modalities.

Surgical innovations

Performing surgeries and getting a skilled hand over the procedure is the ultimate purpose of any budding surgeon, and in this process, years and years of practice are required, along with all the expensive setup and proper patient availability. In this practice, the surgeon has to undergo several surgical procedures, first as an assistant and eventually as a presiding surgeon. Sometimes mistakes occur during this training period, which is at the cost of the quality of the patient’s life or a life-and-death scenario. Many times the risk-to-profit ratio of such procedures is far more than anticipated as multiple factors such as teaching quality, infrastructure, surgeon skill, and many more are of concern while a surgeon is in training. Thus to make this training more user-friendly, the induction of metaverse provisions can be a promising option. Teaching and learning experiences in the surgical field can be enhanced via simulations and avatar building, which is very similar to what it is in Fortnite (Epic Games, Inc., Cary, North Carolina, United States) [27]. Traditional pictorial representation includes graphs and pie diagrams in 2D, but with 3D animation and VR, even the patient can visualize his/her condition better and have an opinion of their own [28].

An excellent example is that in 2019, an intelligent operation theatre complex was constructed in Seoul National University (SNU) Bundang Hospital, Seongnam, South Korea, which consisted of a six-lens VR camera and projection system that could shoot images in all 360° dimensions [29]. It helped the user to see intricate structures like lymph nodes, blood vessels, nerves, microscopic structures and pathologies without actually opening the patient’s body virtually right there in the operation theatre complex. Besides conventional VR and augmented reality, extended reality, also called XR, is more suitable for better results. XR is an advanced version, more like a combination of all the metaverse technologies, from VR to mixed reality and augmented reality [30].

The most crucial part of any surgical management is diagnosis. These days, it is mainly done with investigations, imaging and biochemical tests. In the case of virtual reality induction, places like virtual hospitals are being constructed on a targeted basis to contain the new methodologies of healthcare delivery systems with many possibilities such as diagnostics, preventive medicine, and educational domain [31].

In the case of surgical dentistry, a promising advancement has been made by introducing haptic gloves, via which the students can feel the virtual structure while performing delicate procedures. Significant improvement is seen in the technique with enough practice [32,33]. With this provision, even dentistry will evolve shortly by taking inspiration from medical practices in their metaverse expeditions [34]. Revolutionary changes in medical schools and surgical and interventional training are seen within the metaverse [35]. If diagnostics are concerned, patients would be able to monitor his/her blood pressure, heart rate and glucose levels virtually and even record an electrocardiogram via a particular distant 12 lead ECG device [36].

## Conclusions

Metaverse is a vast concept and is still under research. Every day new advances are made to improve the results of metaverse modalities. Even with all the technological advancements, it still has the capability to expand even more in all directions. In the case of medicine, similar traits can be seen. After considering all the pros and cons of the metaverse as well as the available data around it, the pros are of greater significance. Furthermore, even the cons provide scope for improvement and can be rectified with some further technological development, the establishment of user ethics, regulations and monitoring, and ensuring judicial usage. Thus the induction and usage of virtual reality in significant areas of medicine will only enhance the healthcare service delivery. But the metaverse is still in its initial stages and its full potential is yet to be discovered. Though there still is a grey area where virtual reality introduction can be hostile regarding security and privacy, the overall scenario leans towards the positive side. The cost efficiency of the provisions of metaverse can be neglected for the time being as this is still under the experimentation zone. Once there is enough research done, problems like cost efficiency and global availability can be solved as well. Metaverse can provide a sustainable and dynamic tool for the overall development of the medical sector. Not just the medical sector but the surrounding daughter areas which sustain the working of the medical sector such as finance, human resource development pharmaceuticals can also be benefited from this reform. Extensive research and experimentation are required to perfect the technology. and its usage around the ethical domain is of utmost importance. It still poses the question if it actually represents an emerging technology that will revolutionize our society and healthcare or only an immature condition of the future.

## References

[1] (2022). SPRi. https://spri.kr/posts/view/23165.

[2] Sparkes M (2021). What is a metaverse. New Sci.

[3] Kim S (2020). Metaverse: digital world, world of emerging items. Hwaseong: PlanB Design.

[4] (2022). Mark Zuckerberg Sets Facebook on Long, Costly Path to Metaverse Reality. https://www.wsj.com/articles/mark-zuckerberg-sets-facebook-on-long-costly-path-to-metaverse-reality-11635252726.

[5] Krittanawong C, Rogers AJ, Aydar M, Choi E, Johnson KW, Wang Z, Narayan SM (2020). Integrating blockchain technology with artificial intelligence for cardiovascular medicine. Nat Rev Cardiol.

[6] Shukla M, Lin J, Seneviratne O (2021). BlockIoT: blockchain-based health data integration using IoT devices. AMIA Annu Symp Proc.

[7] Kostick-Quenet K, Mandl KD, Minssen T, Cohen IG, Gasser U, Kohane I, McGuire AL (2022). How NFTs could transform health information exchange. Science.

[8] Subramanian H, Subramanian S (2022). Improving diagnosis through digital pathology: Proof-of-concept implementation using smart contracts and decentralized file storage. J Med Internet Res.

[9] Huh S (2022). Application of computer-based testing in the Korean Medical Licensing Examination, the emergence of the metaverse in medical education, journal metrics and statistics, and appreciation to reviewers and volunteers. J Educ Eval Health Prof.

[10] Smart J, Cascio J, Paffendorf J (2007). Metaverse roadmap: pathways to the 3D web. Ann Arbor.

[11] Kye B, Han N, Kim E, Park Y, Jo S (2021). Educational applications of metaverse: possibilities and limitations. J Educ Eval Health Prof.

[12] (2022). Curiscope. https://www.curiscope.com/.

[13] (2022). Classting AI. https://www.classting.ai/.

[14] Riva G, Wiederhold BK (2022). What the metaverse is (really) and why we need to know about It. Cyberpsychol Behav Soc Netw.

[15] Khatib F, DiMaio F, Cooper S (2011). Crystal structure of a monomeric retroviral protease solved by protein folding game players. Nat Struct Mol Biol.

[16] (2022). Zepeto. https://zepeto.me/..

[17] (2022). Roblox. https://www.roblox.com.

[18] Werner H, Ribeiro G, Arcoverde V, Lopes J, Velho L (2022). The use of metaverse in fetal medicine and gynecology. Eur J Radiol.

[19] Lee J, Kwon KH (2022). Future value and direction of cosmetics in the era of metaverse. J Cosmet Dermatol.

[20] Xi N, Chen J, Gama F, Riar M, Hamari J (2022). The challenges of entering the metaverse: an experiment on the effect of extended reality on workload. Inf Syst Front.

[21] Mesko B (2022). The promise of the metaverse in cardiovascular health. Eur Heart J.

[22] Ifdil I, Situmorang DD, Firman F, Zola N, Rangka IB, Fadli RP (2022). Virtual reality in metaverse for future mental health-helping profession: an alternative solution to the mental health challenges of the COVID-19 pandemic. J Public Health (Oxf).

[23] Situmorang DD (2020). Online/cyber counseling services in the COVID-19 outbreak: are they really new?. J Pastoral Care Counsel.

[24] Situmorang DD (2022). "When the first session may be the last!": a case report of the implementation of "rapid tele-psychotherapy" with single-session music therapy in the COVID-19 outbreak. Palliat Support Care.

[25] Parsons TD (2012). Virtual simulations and the second life metaverse: paradigm shift in neuropsychological assessment. IGI Global.

[26] Aalbers S, Fusar-Poli L, Freeman RE (2017). Music therapy for depression. Cochrane Database Syst Rev.

[27] (2022). Fortnite. https://www.epicgames.com/fortnite/en-US/home.

[28] Skalidis I, Muller O, Fournier S (2022). CardioVerse: the cardiovascular medicine in the era of metaverse. Trends Cardiovasc Med.

[29] Koo H (2021). Training in lung cancer surgery through the metaverse, including extended reality, in the smart operating room of Seoul National University Bundang Hospital, Korea. J Educ Eval Health Prof.

[30] Andrews C, Southworth MK, Silva JN, Silva JR (2019). Extended reality in medical practice. Curr Treat Options Cardiovasc Med.

[31] (2022). "Health City" - Aimedis Publishes Health-Metaverse and Will Soon Open the First Clinic In It. https://finance.yahoo.com/news/health-city-aimedis-publishes-health-174400175.html.

[32] Perry S, Bridges SM, Burrow MF (2015). A review of the use of simulation in dental education. Simul Healthc.

[33] Huang TK, Yang CH, Hsieh YH, Wang JC, Hung CC (2018). Augmented reality (AR) and virtual reality (VR) applied in dentistry. Kaohsiung J Med Sci.

[34] Kurian N, Cherian JM, Varghese KG (2022). Dentistry in the metaverse. Br Dent J.

[35] Suh W, Ahn S (2022). Utilizing the metaverse for learner-centered constructivist education in the post-pandemic era: an analysis of elementary school students. J Intell.

[36] Skalidis I, Muller O, Fournier S (2022). The metaverse in cardiovascular medicine: applications, challenges, and the role of non-fungible tokens. Can J Cardiol.

